# Neural coding strategies for extracting motion estimates from electrosensory contrast

**DOI:** 10.1186/1471-2202-15-S1-P185

**Published:** 2014-07-21

**Authors:** Stephen E Clarke, Richard Naud, André Longtin, Leonard Maler

**Affiliations:** 1 Department of Cellular and Molecular Medicine, University of Ottawa, Ottawa, Ontario, Canada, K1H 8M5; 2 Department of Physics, University of Ottawa, Ottawa, Ontario, Canada, K1N 6N5

## 

Object saliency is based on the relative local-to-background contrast in the physical signals that underlie perceptual experience. Extracting meaningful stimulus representations from these contrast patterns constitutes a serious inverse problem for neural coding, where many different stimulus features map into a scalar firing rate. This decoding problem is even more acute for neurons whose dynamics are characterized by fixed timescales, which produce distinct firing rate responses to different temporal parameterizations of a stimulus [1]. By studying how looming object distance is extracted from electrosensory contrast, we demonstrate that power law adaptation in the primary electroreceptor afferents of *A. leptorhynchus* transforms the firing rate, yielding a code for stimulus intensity (distance) that is invariant to the rate of change of intensity with respect to time (speed). As such, estimates of both object distance and approach speed are effectively parsed into distinct measures of an electroreceptor afferent’s firing rate [1].

Despite its important role in neural coding, adaptation suffers a serious caveat for encoding natural inputs: it generates skewed responses when signal intensity changes from increasing to decreasing (or vice versa). As predicted by generic models of spike-frequency adaptation, we report a skew in the electroreceptor afferent firing rate upon motion reversal, introducing further ambiguity into the estimation of object distance from electrosensory contrast [2]. The electrosense compensates for the skewed firing rate by combining the activity of downstream ON and OFF cells into a population rate code characterized by paradoxical responses to local contrast. Under both positive and negative contrast conditions, motion reversal induces a coding switch between ON and OFF cells, whose combined instantaneous firing rates cooperatively produce a symmetric representation of object motion. Whereas the firing rates of the individual ON and OFF cells convey scalar information, such as object distance, their sequential activation over longer timescales encode changes in motion direction.

## References

[B1] Clarke SE Naud R Longtin A Maler L Speed-invariant encoding of looming object distance requires power law spike rate adaptation P.N.A.S USA 2013 15 33 13624 13629 10.1073/pnas.1306428110 PMC3746935 23898185

[B2] Clarke SE Longtin A Maler L A neural code for looming and receding motion is distributed over a population of electrosensory ON and OFF contrast cells J. Neurosci 2014 15 16 5582 5594 10.1523/JNEUROSCI.4988-13.2014 24741048 PMC6608223

